# Growth hormone peak modifies the effect of BMI on increased systolic blood pressure in children with short stature

**DOI:** 10.1038/s41598-019-44299-9

**Published:** 2019-05-27

**Authors:** Yanying Li, Yanhong Zhang, Mei Zhang, Wanling Yang, Baolan Ji, Hui Pan, Bo Ban

**Affiliations:** 1 Qingdao University, Qingdao, 266071 Shandong China; 2 Department of Endocrinology, Affiliated Hospital of Jining Medical University, Jining Medical University, 89 Guhuai Road, 272029 Jining, Shandong China; 3 Chinese Research Center for Behavior Medicine in Growth and Development, 89 Guhuai Road, 272029 Jining, Shandong China; 4 Hong Kong University, 999077 Hong Kong, China; 5 Key Laboratory of Endocrinology of National Health and Family Planning Commission, Department of Endocrinology, Peking Union Medical College Hospital, Chinese Academy of Medical Science and Peking Union Medical College, 100730 Beijing, China

**Keywords:** Cardiology, Growth disorders, Cardiology, Growth disorders

## Abstract

Blood pressure (BP), especially systolic BP (SBP), is higher in adult growth hormone deficiency (AGHD) patients than in normal controls. Additionally, obesity is a known risk factor for hypertension, and growth hormone deficiency (GHD) is an important cause of short stature. For children with GHD, attention has been directed solely towards height. Few studies have assessed its potential impact on BP. Here, we investigated the effect of body mass index standard deviation score (BMISDS) on BP in children with short stature. This study included 736 children with short stature divided into two groups based on peak growth hormone (GH) level in GH provocation tests [severe GHD (SGHD) group = 212 children; non-SGHD group = 524 children]. We found that SBP was significantly higher in the SGHD group than in the non-SGHD group (*p* = 0.045). Additionally, there was a significant positive association between BMISDS and SBP in the SGHD group (β = 3.12, 95% CI: 1.40–4.84, *p* < 0.001), but no association between these variables was observed in the non-SGHD group. Thus, SGHD patients had a higher SBP than non-SGHD patients. BMISDS is a significant factor for higher SBP in SGHD patients but not in non-SGHD patients.

## Introduction

Adult growth hormone deficiency (AGHD) is associated with a metabolic profile similar to that of metabolic syndrome, which is defined by the clustering of obesity, dyslipidemia, glucose intolerance, and hypertension^1^. All these factors may accelerate atherosclerosis and promote higher cardiovascular morbidity, and untreated AGHD is widely accepted to lead to cardiovascular diseases (CVDs), whereas growth hormone (GH) treatment has beneficial metabolic effects in these patients^2^. On the other hand, the situation in children is less clear, as few studies have investigated metabolic abnormalities and their association with CVD in children. A recent review by De Leonibus C suggested that the development of atheromatous plaques begins early in childhood in the context of GH deficiency (GHD)^3^. Thus, understanding the risk factors for GHD in children is important for preventing disease and reducing morbidity caused by GHD.

In the general population, obesity is a known risk factor for hypertension. Obesity contributes to the development of hypertension via several interconnected and complex pathways, such as inflammation, oxidative stress, insulin resistance, and neurohormonal and humoral dysfunction^4^. Obesity is the excessive accumulation or expansion of adipose tissue. Population studies revealed that GH influences body composition^5,6^ and that GHD is associated with weight gain and a higher body mass index (BMI) in children and adults^7^. Basic research further showed that GH could reduce adipose tissue mass by regulating adipokine secretion, cellular senescence, the immune cell profile, angiogenesis, and lipid droplet formation^8^.

Blood pressure (BP), especially systolic BP (SBP), was reported to be higher in AGHD patients than in normal controls^9,10^. However, these data were obtained from heterogeneous cohorts of adult patients with GHD of varying etiologies and durations. How BP changes in children with GHD is unknown, and the relationship between BMI and BP in children with GHD remains unclear.

GHD is a common cause of linear growth restriction in children, and the diagnosis of GHD mainly relies on clinical manifestations combined with the GH provocation test. There are significant differences in body composition and metabolic traits between patients with severe GHD (SGHD) and the general population^11^. According to the criteria for SGHD^10–12^, this study defined SGHD as a GH peak <5 ng/ml. In this study, we sought to investigate the relationship between BMI standard deviation score (BMISDS) and SBP in children with SGHD.

## Results

### Clinical and laboratory characteristics of the subjects

Seven hundred thirty-six patients aged 10.2 ± 3.5 years, including 516 boys and 220 girls, were enrolled in this study. The demographic, anthropometric, and clinical characteristics of the 736 patients stratified according to GH peak in the GH provocation test are shown in Table 1. Two hundred twelve patients were assigned to the SGHD group based on a GH peak lower than 5 ng/ml, and the other 524 patients were assigned to the non-SGHD group. The patients in the SGHD group showed higher SBP (*p* = 0.035), weight (*p* < 0.001), BMI (*p* < 0.001), BMISDS (*p* < 0.001), insulin-like growth factor-1 standard deviation score (IGF-1SDS) (*p* = 0.002), triglyceride (TG) (*p* = 0.023), total cholesterol (TC) (*p* < 0.001) and low-density lipoprotein cholesterol (LDL-C) (*p* = 0.008) than the non-SGHD group. However, there was no significant difference between groups in diastolic BP (DBP), sex, age, height, height standard deviation score (HtSDS), IGF-1, insulin-like growth factor-binding protein-3 (IGFBP-3), fasting plasma glucose (FPG), high-density lipoprotein cholesterol (HDL-C) or bone age (BA).

**Table 1:** Demographic characteristics and biochemical values of the subjects grouped by GH peak.

Variables	GH peak		*P*-value
	GH peak <5 ng/ml	GH peak ≥5 ng/ml	
N	212	524	
Sex [N(%)]			0.340
Male	154 (72.6%)	362 (69.1%)	
Female	58 (27.4%)	162 (30.9%)	
Age (years)	10.4 ± 3.2	10.1 ± 3.6	0.193
Height (cm)	127.2 ± 16.5	125.0 ± 18.1	0.129
HTSDS	−2.5 ± 0.6	−2.6 ± 0.6	0.075
Weight (kg)	30.6 ± 12.1	26.8 ± 10.3	<0.001
BMI (kg/m^2^)	18.1 ± 3.7	16.5 ± 2.7	<0.001
BMISDS	0.2 ± 1.2	−0.4 ± 1.1	<0.001
BMISDS subgroup			0.183^b^
BMISDS≥2	10 (4.8%)	13 (2.5%)	
−2<BMISDS<2	189 (91.3%)	478 (91.9%)	
BMISDS≤−2	8 (3.9%)	29 (5.6%)	
SBP (mmHg)	105.3 ± 12.1	102.8 ± 11.0	0.048
DBP (mmHg)	63.3 ± 8.4	62.6 ± 9.0	0.327
GH peak (ng/ml)^a^	3.5 (0.0–11.6)	9.5 (3.1–35.7)	<0.001
IGF-1 (ng/ml)^a^	148 (91.2–227.0)	159.0 (88.9–264.0)	0.166
IGF-1SDS	−1.3 ± 1.2	−1.0 ± 1.3	0.002
IGFBP3 (µg/ml)	4.2 ± 1.4	4.4 ± 1.4	0.114
TC (mmol/L)	4.0 ± 0.7	3.8 ± 0.7	<0.001
TG (mmol/L)^a^	0.8 (0.5–0.9)	0.6 (0.5–0.8)	0.023
LDL-C (mmol/L)	2.1 ± 0.5	2.0 ± 0.6	0.009
HDL-C (mmol/L)	1.4 ± 0.3	1.4 ± 0.3	0.129
FPG (mmol/L)	4.7 ± 0.7	4.8 ± 0.8	0.083
BA (years)	8.7 ± 3.5	8.2 ± 3.8	0.124

GH peak: growth hormone peak after provocation; HTSDS: the standard deviation score of height; BMI: body mass index; BMISDS: the standard deviation score of BMI; SBP: systolic blood pressure; DBP: diastolic blood pressure; GH peak: growth hormone peak after provocation; IGF-1: insulin-like growth factor-1; IGF-1 SDS: the standard deviation score of IGF-1; IGFBP-3: insulin-like growth factor-binding protein-3; TC: total cholesterol; TG: triglyceride; HDL-C: high-density lipoprotein cholesterol; LDL-C: low-density lipoprotein cholesterol; FPG: fasting plasma glucose; BA: bone age. ^a^Abnormally distributed variables; ^b^*P* value for comparison of BMISDS distribution between two groups.

### Associations between anthropometric (SBP and DBP) and biochemical variables

Univariate analysis was performed to assess the relationship between each variable and BP. As shown in Table 2, age, height, weight, BMI, BMISDS, IGF-1, IGF-1SDS, IGFBP3, BA, height and TG were significantly positively associated with SBP. It was also shown that age, height, weight, BMI, IGF-1, IGF-1SDS, IGFBP3, and BA were significantly positively associated with DBP, and a significant negative association was found between HtSDS and DBP (*p* = 0.042).

**Table 2:** Association for variables and BP (include SBP and DBP) evaluated by univariate analysis.

	SBP (mmHg)		DBP (mmHg)	
	β (95% CI)	*P*-value	β (95% CI)	*P-*value
SEX				
Male (n = 516)	Reference		Reference	Reference
Female (n = 220)	−3.74 (−5.59, −1.90)	<0.001	−1.16 (−2.56, 0.25)	0.106
Age (year)	1.58 (1.36, 1.79)	<0.001	0.87 (0.70, 1.05)	<0.001
Height (cm)	0.31 (0.27, 0.35)	<0.001	0.16 (0.13, 0.19)	<0.001
HTSDS	−0.47 (−1.86, 0.92)	0.510	−1.09 (−2.13, −0.04)	0.042
Weight (kg)	0.56 (0.49, 0.63)	<0.001	0.26 (0.20, 0.31)	<0.001
BMI (kg/m^2^)	1.58 (1.32, 1.83)	<0.001	0.65 (0.44, 0.85)	<0.001
BMISDS	1.70 (1.00, 2.41)	<0.001	0.49 (−0.05, 1.03)	0.077
GH peak (ng/ml)	0.07 (−0.08, 0.21)	0.356	0.05 (−0.06, 0.16)	0.335
TG (mmol/L)	4.01 (1.39, 6.62)	0.003	1.70 (−0.29, 3.69)	0.094
TC (mmol/L)	0.17 (−1.08, 1.42)	0.790	0.06 (−0.89, 1.01)	0.904
HDL (mmol/L)	−0.03 (−0.18, 0.11)	0.646	−0.02 (−0.14, 0.09)	0.689
LDL (mmol/L)	0.34 (−0.46, 1.14)	0.406	−0.08 (−0.68, 0.53)	0.803
FPG (mmol/L)	0.09 (−0.11, 0.28)	0.374	−0.01 (−0.15, 0.14)	0.977
IGF-1 (ng/ml)	0.04 (0.03, 0.04)	<0.001	0.02 (0.01, 0.02)	<0.001
IGF-1SDS	1.93 (1.25, 2.61)	<0.001	0.71 (0.18, 1.25)	0.009
IGFBP3 (µg/ml)	2.62 (1.93, 3.30)	<0.001	1.17 (0.64, 1.70)	<0.001
BA (year)	1.45 (1.24, 1.65)	<0.001	0.81 (0.64, 0.97)	<0.001

SBP: systolic blood pressure; DBP: diastolic blood pressure; HTSDS: the standard deviation score of height; BMI: body mass index; BMISDS: the standard deviation score of BMI; GH peak: growth hormone peak after provocation; IGF-1: insulin-like growth factor-1; IGF-1 SDS: the standard deviation score of IGF-1; IGFBP-3: insulin-like growth factor-binding protein-3; TC: total cholesterol; TG: triglyceride; HDL-C: high-density lipoprotein cholesterol; LDL-C: low-density lipoprotein cholesterol; FPG: fasting plasma glucose; BA: bone age. p < 0.05 is considered to be statistically significant.

### Effect of BMISDS on SBP in different GH peak groups

Given the above results showing that BMISDS was significantly associated with SBP (β = 1.70, 95% CI 1.00–2.41; *p* < 0.001) (Table 2), we generated a linear regression model with different GH peak groups. As shown in Table 3, the crude model revealed a significant positive association between BMISDS and SBP in the SGHD group (β = 3.57, 95% CI 2.30–4.85; *p* < 0.001), but there was no association in the non-SGHD group (β = 0.75, 95% CI −0.12–1.62; *p* = 0.091). To further confirm the findings, we adjusted the models for TG, TC, HDL-C, LDL-C, FPG, IGF-1SDS and IGFBP3 and found an independent association between BMISDS and SBP in the SGHD group (β = 3.12, 95% CI: 1.40–4.84, *p* < 0.001) but not in the non-SGHD group (β = 0.94, 95% CI: −0.08, 1.95, *p* = 0.071). Furthermore, a test of the interaction between GH peak and BMISDS was statistically significant (*p* = 0.001).

**Table 3:** Effect of BMISDS on SBP based on GH peak.

Group	Crude Model		*P* value for interaction	Adjusted Model		*P* value for interaction
	β (95% CI)	*P* value		β (95% CI)	*P* value	
GH peak <5 ng/ml (n = 212)	3.57 (2.30, 4.85)	<0.001	<0.001	3.12 (1.40, 4.84)	<0.001	0.001
GH peak ≥ 5 ng/ml (n = 524)	0.75 (−0.12, 1.62)	0.091		0.94 (−0.08, 1.95)	0.071	

*P* value for interaction: interaction of GH peak and BMISDS on systolic blood pressure. Adjustment variables: triglyceride, total cholesterol, high-density lipoprotein cholesterol, low-density lipoprotein cholesterol, fasting plasma glucose, the standard deviation score of IGF-1; insulin-like growth factor-binding protein-3; *P* < 0.05 is considered to be statistically significant.

## Discussion

In this retrospective cross-sectional study, we analyzed the association between BMISDS and SBP in children with short stature. The results showed that SBP was significantly higher in the SGHD group than in the non-SGHD group. Further regression analysis showed that BMISDS was independently associated with SBP in the SGHD group, but there was no evidence of such an association in the non-SGHD group.

Previous studies have documented that GHD patients have a higher prevalence of obesity than the general population. In the present study, we found that patients with SGHD had a higher BMI than those in the non-SGHD group. In addition, according to the diagnostic criteria for childhood obesity^13^, the incidence of obesity was 4.8% in the SGHD group and 2.5% in the non-SGHD group, while the incidence rates of leanness in the SGHD and non-SGHD groups were 3.9% and 5.6%, respectively. Although these differences were not statistically significant, the trend of increased obesity in patients with SGHD was obvious, and the finding is consistent with reports in the literature^10^.

Previous studies have reported that GHD patients have an increased prevalence of hypertension compared with the general population^10,14–16^. However, these results are based on AGHD with different causes and courses. To our knowledge, no similar studies in children with GHD are available. The study presented herein showed that SBP was significantly higher in SGHD children than in non-SGHD children. No difference in DBP was found between the two groups, which is inconsistent with previous findings^9,10^. However, the underlying mechanism requires further exploration.

Obesity is an established risk factor for hypertension^17,18^, which is more than twice as prevalent in obese individuals than in nonobese individuals^19^. BMI is an indicator of obesity. Our study indicated that BMISDS was positively related to SBP by univariate analysis, and further subgroup analysis revealed that SBP was positively related to BMISDS in the SGHD group but not in the non-SGHD group. Moreover, multivariate linear regression analysis demonstrated that BMISDS had an independent effect on SBP in patients with SGHD.

Although the specific mechanism for the relationship between BMISDS and SBP remains unclear, we considered based on our findings that this relationship may differ based on the degree of GHD. A previous study showed that metabolic index did not differ between patients with GH peaks above 7 ng/ml and people with normal GH peaks, while a significant difference existed between patients with SGHD and an unaffected population^11^. Abdominal fat accumulation is a main metabolic feature of GHD, which may lead to abdominal obesity^10^. People with abdominal obesity reportedly have a 2–3-fold higher risk of hypertension^20,21^. Thus, we concluded that increased abdominal fat caused by a higher BMI in patients with SGHD may partly explain the difference in SBP between the SGHD and non-SGHD groups. Given the lack of evidence, related studies should be carried out to explore the potential mechanism.

Some limitations in our study must be noted. First, we did not collect data regarding other potential contributors to SBP, such as diet and family history. We intend to analyze the effects of diet, family history and pubertal state on SBP in a prospective study. Second, due to the cross-sectional design of this study, the present findings showed a positive association between BMISDS and SBP in only children with SGHD. Prospective studies are required to further observe the results after GH treatment.

In conclusion, children with SGHD presented an elevated SBP compared with non-SGHD children, and in the SGHD group, SBP was positively associated with BMISDS, suggesting that CVD risk factors such as hypertension and obesity should be of concern in children with SGHD.

## Subjects and Methods

### Study subjects

**Figure 1:**
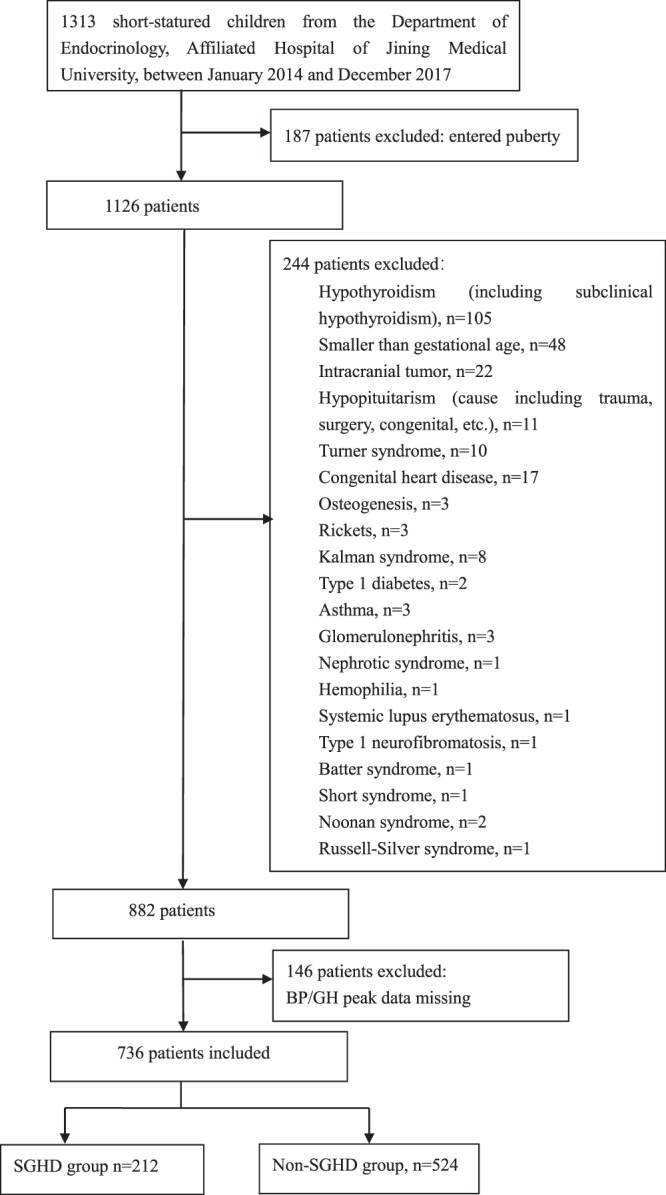
Flow chart of study participants SGHD: severe growth hormone deficiency; non-SGHD: non-severe growth hormone deficiency group.

We retrospectively reviewed the medical records of children with short stature from the Department of Endocrinology, Affiliated Hospital of Jining Medical University, between January 2014 and December 2017. The subjects were selected based on the following inclusion criteria: short stature, which was defined as the height of an individual being below the third percentile of the corresponding population for a given age, sex and ethnic group; normal weight and length at birth; and normal prepuberty state. The exclusion criteria included the presence of chronic diseases, chromosomal abnormalities, skeletal dysplasia, genetic metabolic diseases and endocrine system diseases except for GHD, as well as the use of medications that interfere with BP or BMI. Overall, 1313 patients were available during the study period, 736 of whom were eligible for our study and were included, as described in the flow chart (Fig. 1). All enrolled patients were examined for GHD and idiopathic short stature (ISS) based on the GH peak level in provocation tests. A GH peak ≥10 ng/ml indicated a diagnosis of ISS, and a GH peak <10 ng/ml indicated a diagnosis of GHD which included partial GHD (GH peak ≥5 ng/ml) and complete GHD (GH peak <5 ng/ml); all enrolled patients were subdivided into the non-SGHD (partial GHD and ISS; GH peak ≥5 ng/ml) and SGHD groups (complete GHD; GH peak <5 ng/ml).

The study was approved by the Human Ethics Committee of the Affiliated Hospital of Jining Medical University (Shandong, China), and all methods were performed in accordance with the guidelines of the Declaration of Helsinki. All of the families of the patients were informed of the aims of the study, and written informed consent was obtained from the parents of the patients.

### Anthropomorphic measurements

Height and weight were measured by a designated individual using the same measuring instrument in the morning with allowable error ranges of 0.1 cm and 0.1 kg, respectively. BP was measured in the seated position by one of two pretrained physicians using an electronic sphygmomanometer with an appropriately sized cuff for the arm of a child, and the average of three measurements was used for analysis. HtSDS was calculated based on the normal range of Chinese children^22^. BMI was calculated as the ratio between body weight in kilograms and height in meters squared. The stage of puberty was assessed by physical examination according to Tanner staging^23^; prepuberty was defined^24^ as a testicular volume less than 4 ml and no pubic hair in boys and as no breast development or pubic hair in girls.

### Laboratory measurements

To assess GH secretion, Ldopa (Levodopa Tablets, He Feng, Guang Xi, China; dose: 500 mg for patients weighing more than 30 kg and 250 mg for those weighing less than 30 kg) and insulin (Insulin Injection, Wan Bang, Jiang Su, China; 0.1 U/kg) were administered orally or subcutaneously after overnight fasting. Blood samples were collected at 0, 30, 60, 90, and 120 min after administration to obtain serum GH concentrations at each time point. GH was measured using a chemiluminescence method (ACCESS2, Beckman Coulter; USA) with an analytical sensitivity of 0.010 µg/L. Serum IGF-1 and IGFBP-3 levels were measured by the chemiluminescence immunometric method (DPCIMMULITE 1000 analyzer, SIEMENS, Germany); the intra- and interassay coefficients of variation (CVs) were 3.0% and 6.2% for IGF-1 and 4.4% and 6.6% for IGFBP-3. Measures of liver function (including alanine aminotransferase (ALT), AST, and gamma-glutamyl transferase (GGT)) and kidney function (including Cr, blood urea nitrogen (BUN), and UA), lipid profiles (including TC, HDL-C, LDL-C, and TG), and FPG were obtained by a biochemical autoanalyzer (Cobas c 702, Roche; Shanghai, China). Measures of thyroid function, including free T3 (FT3), free T4 (FT4), thyroid-stimulating hormone (TSH), gonadotropin, cortisol, and adrenocorticotropic hormone (ACTH), were determined by a luminescence immunoassay system (Cobas c 602, Roche; Shanghai, China). IGF-1SDS and BMISDS were calculated according to a previous study^22,25^.

### Statistical analysis

All statistical analyses were performed with R statistical software (https://www.r-project.org;) and EmpowerStats (http://www.empowerstats.com, X&Y Solutions, Inc. Boston MA). Normally distributed variables are expressed as the mean ± standard deviation (SD); non-normally distributed variables are presented as the median (quartile). Categorical data are expressed as percentages. To compare differences between two groups, Student’s t-test was used for normally distributed variables, the Kruskal-Wallis test was used for non-normally distributed variables, and the chi-square test and Fisher’s exact test were used for categorical variables. A univariate model was used to examine whether BMISDS and other anthropometric and biochemical variables were associated with SBP and DBP. Then, the association between BMISDS and SBP in each subgroup defined by GH peak was estimated using a multivariate linear regression model adjusted for pertinent variables, and the interaction of GH peak with BMISDS was tested. A two-tailed *P* value ≤0.05 was considered to indicate statistical significance in all analyses.
